# A poor perspective of self weight significantly increases adverse outcomes in non-alcoholic fatty liver disease (NAFLD)

**DOI:** 10.3389/fmed.2022.977552

**Published:** 2022-09-28

**Authors:** Clarissa Elysia Fu, Cheng Han Ng, Nicholas W. S. Chew, Zane En Qi Heng, Yip Han Chin, Jingxuan Quek, Wen Hui Lim, Jieling Xiao, Kai En Chan, Darren Jun Hao Tan, Caitlyn Tan, Sitong Zhang, Teng Kiat Koh, Benjamin Nah, Yock Young Dan, Nicholas Syn, Mohammad Shadab Siddiqui, Arun J. Sanyal, Mazen Noureddin, Mark Muthiah

**Affiliations:** ^1^Yong Loo Lin School of Medicine, National University of Singapore, Singapore, Singapore; ^2^Department of Cardiology, National University Heart Centre, National University Hospital, Singapore, Singapore; ^3^Division of Gastroenterology and Hepatology, Department of Medicine, National University Hospital, Singapore, Singapore; ^4^National University Centre for Organ Transplantation, National University Health System, Singapore, Singapore; ^5^Division of Gastroenterology, Hepatology and Nutrition, Department of Internal Medicine, Virginia Commonwealth University, Richmond, VA, United States; ^6^Houston Research Institute, Houston Liver Institute, Houston, TX, United States

**Keywords:** weight perception, NAFLD, non-alcoholic fatty liver disease, NAFLD outcomes, mortality

## Abstract

**Background:**

Non-alcoholic fatty liver disease (NAFLD) is prevalent amongst overweight and obese individuals, and weight loss remains the main mode of treatment for NAFLD patients. Weight perception plays a key role in the efficacy of such treatment. The current study aims to investigate the prevalence, associating factors and implications of poor weight perception amongst such individuals.

**Methods:**

An analysis was done on data collected from NHANES between 1999 and 2018. Comparison was made between NAFLD individuals with and without poor weight perception in terms of prevalence, associated characteristics, and clinical outcomes. Multivariate analysis was used to compare effect size of adverse events associated with NAFLD individuals with poor weight perception.

**Results:**

Of the 12,170 NAFLD patients, 19.2% (CI: 18.5 to 19.9%) had poor weight perception. Poor weight perception was significantly associated with lower education levels, reduced levels of exercise and unhealthier lipid profiles. There was an increased risk in all-cause mortality (HR: 1.18, CI: 1.00 to 1.38, *p* = 0.047), cardiovascular disease mortality (SHR: 1.33, CI: 1.03 to 1.71, *p* = 0.026), major adverse cardiovascular events (OR: 1.21 CI: 1.10 to 1.32, *p* < 0.001), and advanced fibrosis (OR: 1.30, CI: 1.03 to 1.64, *p* = 0.025) for individuals with poor weight perception.

**Conclusion:**

This study highlights the positive association between appropriate weight perception and better outcomes in individuals with NAFLD. Poor weight perception increased the risk of adverse events and decreased inclination toward seeking weight loss treatment. Greater emphasis should be placed on dealing with weight perception in individuals with NAFLD for better treatment outcomes.

## Introduction

Non-alcoholic fatty liver disease (NAFLD) is among the commonest cause of liver disease globally affecting about 25 to 33% of the global population (1, 2). NAFLD is a consequent by-product of systemic metabolic dysfunctions closely associated with type 2 diabetes mellitus, hypertension, dyslipidemia, and obesity (3–6) and ranges in severity from simple steatosis to non-alcoholic steatohepatitis (NASH). The presence of NAFLD however is associated with an increased risk of adverse events such as liver-related complications, metabolic and cardiovascular diseases (7–10). Obesity and NAFLD are closely associated, whereby overweight and obese individuals are at an increased risk of NAFLD and 50.7% of them having NAFLD (11, 12). Excess visceral fat results in adipocyte dysfunction and insulin resistance, leading to higher levels of lipolysis. Consequently, there will be an increase in circulating free fatty acids, which ultimately contribute to the characteristic excessive intrahepatic fat accumulation in NAFLD (13). The presence of obesity is closely associated with NAFLD, with many shared pathogenesis pathways including dysregulation of adipokines, lipotoxicity, endoplasmic reticulum, and oxidative stress (14–16).

In the absence of suitable pharmacological treatments for NAFLD (17), behavioral modification with the goal of weight loss through dietary adjustments and physical exercise remains the main strategy for patients affected by NAFLD (18–20). Weight management forms an essential cornerstone in NAFLD where previous studies have shown that individuals with weight loss were significantly associated with improvements in histologic NAFLD activity score (21). Despite this, previous studies have reported that only 53.9% of people with NAFLD have the intention to lose weight (22). A key factor affecting weight loss relates to their self-perception of weight. Individuals who perceived themselves to be overweight are four times more likely to have intentions of weight loss (22). Yet, it remains to be seen if poor weight perception significantly alters the longitudinal prognosis of NAFLD patients and an understanding of this will allow for better risk stratification and prognostication as well as patient management. Hence this study aims to investigate the prevalence, associating factors and implications of poor weight perception amongst patients with NAFLD through analysis of National Health and Nutrition Examination Survey (NHANES) database with supplementary longitudinal data from the National Death Index (NDI).

## Methods

### Study population

Data for this study was collected from NHANES between 1999 and 2018 and used for analysis. NAFLD patients who were overweight or obese were included in this study. Briefly, the NHANES study is a health database consisting of responses to a stratified and clustered sampled national health survey which comprise of a thorough interview, medical examination and laboratory assessments done on individuals representative of the general, non-institutionalized United States population. The full methodology of data collection was published previously (23). Linking of the NHANES dataset to the death certificates derived from the NDI was conducted to facilitate mortality analysis in this study. In this present analysis, information used has been publicly published by National Center for Health Statistics (NCHS) and is anonymous in nature, thus exempted from review by the Institutional Review Board. Demographic variables (age, gender, ethnicity, body mass index [BMI], income, education levels), laboratory variables (low-density lipoprotein [LDL] cholesterol, direct high-density lipoprotein [HDL] cholesterol, total cholesterol, triglyceride, fasting blood glucose, glycated hemoglobin [HbA1c]) and past medical history (diabetes, hypertension, obesity) were collected (24).

### Definitions

In this analysis, self-perception of one's weight was determined by administering a questionnaire with two options of whether they consider themselves to be (1) “overweight” or (2) “underweight or about right.” Patients were considered to have “poor weight perception” if they determine themselves to be “underweight or about right” despite being categorized as overweight or obese via BMI. On the other hand, individuals who correctly identified themselves to be “overweight” are “without poor weight perception.” Patients were considered overweight if they had BMI of 25.0–30.0 kg/m^2^ in Caucasians or 23–27.5 kg/m^2^ in Asian populations. Obese individuals were identified if they had a BMI of ≥30 kg/m^2^ for Caucasians or ≥27.5 kg/m^2^ in Asian populations. The diagnosis for NAFLD was made in accordance to the American Association for the Study of Liver Disease (AASLD) guidelines for NAFLD (3). The criteria states that there has to be (1) Evidence of hepatic steatosis using fatty liver index (FLI)/United States Fatty Liver Index (US-FLI), (2) Lack of substantial alcohol consumption ( ≤ 21 drinks/week in men, ≤ 14 drinks/week in women). An FLI ≥ 60 (25) or US-FLI ≥ 30 (26) indicates the presence of hepatic steatosis which has a corresponding Area Under Receiver Operating Characteristics Curve (AUROC) of 0.834 (27). Quantification of fibrosis in the liver was examined using Fibrosis-4 (FIB-4) Index Score, where a score of ≥2.67 was defined as advanced fibrosis. Hypertension (HTN) was identified for any individual with systolic blood pressure ≥140, diastolic blood pressure ≥90 or the use of antihypertensive therapy (28). The criterion for diabetes is as follows: (1) A physician diagnosis of diabetes, (2) fasting plasma glucose ≥7 mmol/l and HbA1c ≥ 6.5%, (3) treatment with any oral hypoglycemic agents or insulin. Major adverse cardiovascular events (MACE) include occurrence of heart failure, stroke, myocardial infarction, and cardiovascular disease (CVD) mortality. Exercise perception was determined by administering a survey whereby individuals had to indicate if they were (1) willing to exercise or (2) not willing to exercise.

### Statistical analysis

STATA (16.1) was used to conduct all statistical analysis. The Wilcoxon ranked sum test and Kruskal-Wallis analysis was used to assess continuous variables in two or more groups. Chi-square test was used to assess binary variables and the magnitude of effect was assessed using a generalized linear model with a log link. Odds ratio was used to estimate common events and clinical interpretability. Multivariate analysis, with adjustment for age, gender, ethnicities, BMI and diabetes was done for risk factors. Cox proportional model was used for survival analysis for all-cause mortality while a log-log plot and Schoenfeld residuals were used to assess the violations of proportionality. CVD mortality was assessed using a multivariate competing risk analysis with Fine-Gray subdistribution hazard ratio (SHR) was used. The multivariate model was adjusted for BMI, gender, age, ethnicity, income, exercise, and diabetes mellitus. All univariate and multivariate analysis was conducted with clustering study year to account for heterogeneity between different year of study.

## Results

### Prevalence and factors associated with poor weight perception

A total of 12,170 individuals with NAFLD were included in the analysis and 19.2% (CI: 18.5% to 19.9%) of patients classified themselves as normal weight individuals despite being significantly overweight. The factors that were associated with poor weight perception can be found in Table 1. Factors associated with poor weight perception included an older age (56 vs. 53 years, *p* < 0.001), male gender (67.7% vs. 44.2%, *p* < 0.001) and lower BMI (29.2 vs. 33.6 kg/m^2^, *p* < 0.001). Individuals without hypertension (61.1% vs. 63.5%, *p* = 0.037) or obesity (43.4% vs. 81.2%, *p* < 0.001) were associated with poor weight perception as well. Other factors associated with poor weight perception include higher total cholesterol levels (200 mg/dL vs. 198 mg/dL, *p* < 0.001), higher triglyceride levels (176 mg/dL vs. 155 mg/dL, *p* < 0.001) and lower direct HDL-Cholesterol levels (45 mg/dL vs. 46 mg/dL, *p* = 0.001). It was also noted that there was a statistical difference in HbA1c levels (*p* = 0.005) and total bilirubin levels (*p* < 0.001) in individuals with poor weight perception in comparison to individuals without. With regards to ethnicity, a higher percentage of African American, Mexican American, Hispanic and individuals from other ethnicities were associated with poor weight perception. A smaller proportion of Caucasians were associated with poor weight perception (36.6% vs. 46.5%, *p* < 0.001). Poor weight perception was also associated with lower levels of education, with a higher percentage of individuals who received <9th grade education (24.0% vs. 11.7%, *p* < 0.001) associated with poor weight perception. Individuals with poor weight perception were also significantly less likely to be associated with physical activity (17.3% vs. 83.7%, *p* < 0.001).

**Table 1:** Baseline demographics of NAFLD individuals with and without poor weight perception.

	**With poor weight perception** ** (*n* = 2,329)**	**Without poor weight perception** ** (*n* = 9,841)**	***p*-value**
Age (years)	56 (IQR: 39 to 70)	53 (IQR: 39 to 65)	<0.001^*^
Gender (male)	67.7 (95%CI: 65.8 to 69.6)	44.2 (95%CI: 43.2 to 45.2)	<0.001^*^
BMI (kg/m^2^)	29.2 (IQR: 27.4 to 31.8)	33.6 (IQR: 30.6 to 37.7)	<0.001^*^
Hypertension (%)	61.1 (95%CI: 59.0 to 63.1)	63.5 (95%CI: 62.5 to 64.5)	0.037^*^
Diabetes (%)	23.7 (95%CI: 22.0 to 25.5)	24.3 (95%CI: 23.5 to 25.2)	0.565
Obesity (%)	43.4 (95%CI: 41.4 to 45.4)	81.2 (95%CI: 80.4 to 82.0)	<0.001^*^
Platelet count (1,000 cells/uL)	240 (IQR: 200 to 279)	253 (IQR: 213 to 299)	<0.001^*^
Exercise (%)	17.3 (95%CI: 16.7 to 18.9)	83.7 (95%CI: 81.0 to 84.1)	<0.001^*^
Glycated hemoglobin (%)	5.6 (IQR: 5.3 to 6.1)	5.6 (IQR: 5.3 to 6.1)	0.005^*^
FBG (mmol/L)	5.83 (IQR: 5.38 to 6.66)	5.77 (IQR: 5.37 to 6.55)	0.285
Total bilirubin (umol/L)	10.3 (IQR: 8.55 to 13.7)	10.3 (IQR: 6.84 to 13.7)	<0.001^*^
Total Cholesterol (mg/dL)	200 (IQR: 174 to 232)	198 (IQR: 172 to 226)	<0.001^*^
LDL-Cholesterol (mg/dL)	117 (IQR: 93 to 142)	118 (IQR: 94 to 141)	0.758
Direct HDL-Cholesterol (mg/dL)	45 (IQR: 38 to 54)	46 (IQR: 39 to 55)	0.001^*^
Triglycerides (mg/dL)	176 (IQR: 121 to 262)	155 (IQR: 108 to 227)	<0.001^*^
**Income Level** (%)			<0.001^*^
0–10,000	8.79 (95%CI: 7.61 to 10.1)	5.81 (95%CI: 5.33 to 6.34)	
10,000–25,000	29.2 (95%CI: 27.2 to 31.2)	23.7 (95%CI: 22.9 to 24.7)	
25,000–45,000	27.2 (95%CI: 25.3 to 29.3)	25.8 (95%CI: 24.9 to 26.7)	
45,000–75,000	20.4 (95%CI: 18.6 to 22.2)	23.9 (95%CI: 23.0 to 24.9)	
>75,000	14.4 (95%CI: 13.0 to 16.1)	20.7 (95%CI: 19.8 to 21.6)	
**Ethnicity** (%)			<0.001^*^
Caucasian	36.6 (95%CI: 34.7 to 38.6)	46.5 (95%CI: 45.6 to 47.5)	
African American	22.6 (95%CI: 20.9 to 24.3)	21.4 (95%CI: 20.6 to 22.3)	
Mexican American	23.7 (95%CI: 22.1 to 25.5)	18.0 (95%CI: 17.2 to 18.7)	
Hispanic	9.31 (95%CI: 8.20 to 10.6)	7.82 (95%CI: 7.31 to 8.37)	
Others	7.73 (95%CI: 6.71 to 8.89)	6.25 (95%CI: 5.79 to 6.75)	
**Highest level of education** (%)			<0.001^*^
Less than 9th Grade	24.0 (95%CI: 18.6 to 30.4)	11.7 (95%CI: 9.75 to 13.9)	
9–11th Grade	13.5 (95%CI: 9.42 to 19.0)	13.0 (95%CI: 11.0 to 15.4)	
High School	29.0 (95%CI: 23.1 to 35.7)	31.3 (95%CI: 28.4 to 34.4)	
Some College/AA degree	22.0 (95%CI: 16.8 to 28.3)	31.3 (95%CI: 28.4 to 24.4)	
College Graduate	11.5 (95%CI: 7.76 to 16.7)	18.7 (95%CI: 16.3 to 21.4)	

NAFLD, Non-alcoholic Fatty Liver Disease; BMI, Body Mass Index; FBG, Fasting Blood Glucose; LDL, Low-density lipoprotein; HDL, High-density lipoprotein; IQR, Interquartile range; AA, Associates in Arts; 95%CI, 95% Confidence Interval;

* bolded p-value ≤ 0.05 denotes statistical significance.

### Adverse events associated with poor weight perception

**Figure 1:**
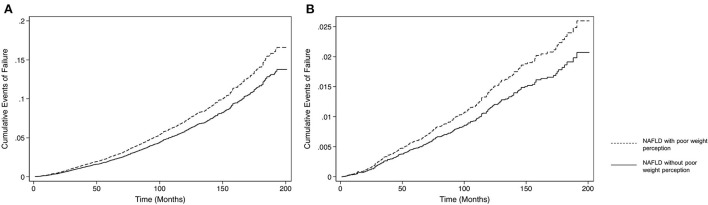
Incidence of all-cause mortality in NAFLD individuals with and without poor weight perception. **(A)** Survival analysis of all-cause mortality. **(B)** Survival analysis of CVD mortality.

An analysis on the adverse events associated with NAFLD individuals poor weight perception in comparison to those without, was done to determine the longitudinal consequences of poor weight perception. The unadjusted effect size of adverse events associated with NAFLD individuals with poor weight perception with reference to those without poor weight perception can be found in Table 2, where NAFLD individuals with poor weight awareness were associated with an increased risk of all-cause mortality (HR: 1.73, CI: 1.52 to 1.97, *p* < 0.001) and CVD mortality (SHR: 1.91, CI: 1.61 to 2.27, *p* < 0.001). These individuals were also significantly associated with an increased risk in MACE (OR: 1.52, CI: 1.35 to 1.70, *p* < 0.001) and advanced fibrosis (OR: 2.08, CI: 1.71 to 2.53, *p* < 0.001). On multivariate analysis adjusted for BMI, gender, age, ethnicity, income, exercise and diabetic status with a clustered analysis on the year of study, NAFLD with poor weight perception were more likely to be associated with advance fibrosis (OR: 1.30, CI: 1.03 to 1.64, *p* = 0.025) and MACE events (OR: 1.21 CI: 1.10 to 1.32, *p* < 0.001). In survival analysis of all-cause mortality, there was a statistically significant increase in all-cause mortality (HR: 1.18, CI: 1.00 to 1.38, *p* = 0.047, Figure 1A) in NAFLD with poor weight perception compared to those without poor weight perception. Similarly, the risk of CVD mortality was significantly higher in NAFLD with poor weight perception (SHR: 1.33, CI: 1.03 to 1.71, *p* = 0.026, Figure 1B).

**Table 2:** Effect size of adverse events in NAFLD individuals with poor weight perception with reference to NAFLD individuals without poor weight perception.

	**Unadjusted logistic regression**		**Multivariate logistic regression** ^**†**^	
	**Effect size (95%CI)**	***p*-value**	**Effect size (95%CI)**	***p*-value**
Overall mortality	1.73 (1.52 to 1.97)	<0.001^*^	1.18 (1.00 to 1.38)	0.047^*^
CVD mortality	1.91 (1.61 to 2.27)	<0.001^*^	1.33 (1.03 to 1.71)	0.026^*^
MACE	1.52 (1.35 to 1.70)	<0.001^*^	1.21 (1.10 to 1.32)	<0.001^*^
Advanced fibrosis	2.08 (1.71 to 2.53)	<0.001^*^	1.30 (1.03 to 1.64)	0.025^*^

NAFLD, Non-alcoholic Fatty Liver Disease; 95%CI, 95% Confidence Interval;

* bolded p-value ≤ 0.05 denotes statistical significance; CVD, Cardiovascular Disease; MACE, Major Adverse Cardiac Events; BMI, Body Mass Index;

† adjusted for BMI, gender, age, ethnicity, income, exercise, diabetes mellitus.

### Adverse events by weight classes

With reference to overweight individuals with NAFLD and without poor weight perception, a subgroup analysis was conducted to examine the effect of poor weight perception on different weight classes (overweight and obese). The unadjusted analysis on MACE, advance fibrosis, all-cause mortality, and CVD mortality can be found in Table 3. On multivariate analysis with BMI, gender, age, ethnicity, income, exercise and diabetic status with a clustered analysis on the year of study, MACE events was significantly associated with both overweight (OR: 1.31, CI: 1.11 to 1.55, *p* = 0.001) and obese NAFLD (OR: 1.65, CI: 1.40 to 1.94, *p* < 0.001) with poor weight perception compared to overweight NAFLD without poor weight perception. Consequently, both overweight (SHR: 1.31, CI: 1.03 to 1.67, *p* = 0.028) and obese NAFLD (SHR: 1.56, CI: 1.15 to 2.11, *p* = 0.004) with poor weight perception were at a higher risk of CVD mortality compared to overweight NAFLD without poor weight perception. In the analysis of advanced fibrosis however, there was no significant increase in association of advance fibrosis in obese NAFLD with poor weight perception compared to overweight NAFLD without poor weight perception (OR: 1.50, CI: 0.903 to 2.50, *p* = 0.117). However, overweight NAFLD with poor weight perception was at an increased odds of advance fibrosis compared to overweight NAFLD without poor weight perception (OR: 1.43, CI: 1.05 to 1.97, *p* = 0.025). Interestingly, there was similar observation made in the analysis of all-cause mortality where overweight NAFLD with poor weight perception were at significantly higher risk of all-cause mortality compared to overweight NAFLD without poor weight perception (HR: 1.30, CI: 1.15 to 1.46, *p* < 0.001). Obese NAFLD was not statistically associated with an increased risk of all-cause mortality compared to overweight NAFLD without poor weight perception (HR: 1.08, CI: 0.789 to 1.48, *p* = 0.643).

**Table 3:** Effect size of adverse events in overweight and obese NAFLD individuals with poor weight perception with reference to overweight NAFLD individuals without poor weight perception.

	**Overweight with poor weight perception**				**Obese with poor weight perception**			
	**Unadjusted logistic regression**		**Multivariate logistic regression** ^**†**^		**Unadjusted logistic regression**		**Multivariate logistic regression** ^**†**^	
	**Effect size (95%CI)**	***p*-value**	**Effect size (95%CI)**	***p*-value**	**Effect size (95%CI)**	***p*-value**	**Effect Size (95%CI)**	***p*-value**
Overall mortality	1.62 (1.42 to 1.86)	<0.001^*^	1.30 (1.15 to 1.46)	<0.001^*^	0.88 (0.54 to 1.45)	0.618	1.08 (0.789 to 1.48)	0.643
CVD mortality	1.69 (1.31 to 2.17)	<0.001^*^	1.31 (1.03 to 1.67)	0.028^*^	1.41 (1.02 to 1.94)	0.035^*^	1.56 (1.15 to 2.11)	0.004^*^
MACE	1.53 (1.31 to 1.78)	<0.001^*^	1.31 (1.11 to 1.55)	0.001^*^	1.43 (1.17 to 1.74)	<0.001^*^	1.65 (1.40 to 1.94)	<0.001^*^
Advanced fibrosis	1.97 (1.44 to 2.71)	<0.001^*^	1.43 (1.05 to 1.97)	0.025^*^	1.29 (0.766 to 2.19)	0.335	1.50 (0.903 to 2.50)	0.117

NAFLD, Non-Alcoholic Fatty Liver Disease; 95%CI, 95% Confidence Interval;

* bolded p-value ≤ 0.05 denotes statistical significance; CVD, Cardiovascular Disease; MACE, Major Adverse Cardiac Events; BMI, Body Mass Index;

† adjusted for BMI, gender, age, ethnicity, income, exercise, diabetes mellitus.

## Discussion

Evidence from current meta-analysis estimates that up to 50.7% of overweight and obese individuals have NAFLD (11). However, not all individuals have accurate perception of their weight, which may affect the motivation for lifestyle modification treatment and in turn disease progression and outcomes. While previous studies have related poor weight perception with poor food choices (29), there remains limited evidence on the effects of poor weight awareness on longitudinal outcomes in NAFLD. The current study therefore shows that up to one fifth of overweight NAFLD individuals do not think themselves to be overweight and the lack thereof in insight conversely affects the motivation to change, evidently seen in the reduction of physical activity. In turn, despite having a lower BMI with significantly less metabolic dysfunction, individuals with poor insights were more likely to be associated with an increase in overall mortality, CVD mortality and MACE events.

Notably, individuals with poor weight perception are significantly associated with unhealthier lipid profiles, with higher total cholesterol and triglyceride levels, and lower HDL-cholesterol levels, likely attributed to the lack of compliance to lifestyle management plans in NAFLD treatment. Interestingly, poor weight perception tends to affect individuals who are overweight compared to obese, as seen whereby the population of individuals with poor weight perception were associated with lower BMI and have lower rates of obesity. With the increasing prevalence of obesity (30), people of larger body types become an increasingly common sight, and this results in visual normalization of larger body types (31), which likely plays a role in the underestimation of weight, especially for individuals with borderline obesity but have not yet approached morbid obesity. Individuals with a lower education background were also more likely to have poor weight perception. This is likely attributed to the lack of opportunity to engage in discussion surrounding the topic of obesity throughout the course of their schooling years. As such these individuals lack of awareness of the adverse effects of obesity and consequently their weight.

Results from the current study shows an increased risk in all-cause mortality, CVD mortality, MACE, and advanced fibrosis in NAFLD individuals with poor weight perception, demonstrating a positive association of accurate weight perception and better clinical outcomes. On further subgroup analysis within the population with poor weight perception, it was revealed that between different weight classes of NAFLD individuals, both overweight and obese individuals have significantly increased risk of MACE. Consequently, both overweight and obese individuals with NAFLD also have an increased risk of CVD mortality. However, it was noted that while overweight individuals, despite having lower levels of metabolic dysfunction, had increased risk of all-cause mortality and advanced fibrosis, and the same was not observed in obese individuals. While one would expect obese individuals to be associated with significantly higher adverse events with larger systemic dysfunction, we believe the observed this anomaly in results could potentially be skewed by the sample size of individuals with poor weight perception in the overweight category which outweighs the number of individuals with obesity.

The results of this analysis highlight the importance centering management efforts into improving weight awareness in NAFLD patients. At present, lifestyle management remains the only effective treatment measure in NAFLD which can additionally serve as a preventive measure for CVD disease (32). However, individuals with poor weight perception were less likely to exercise. Approaching the topic of weight loss to patients can however be difficult in clinical practice and lies a fine balance between improving awareness and being perceived as derogatory or humiliating. Weight is ultimately a sensitive matter particularly amongst the female population, but not addressing the issue can result in an increase in adverse events. However, excessive criticism can result in psychological harm and disengagement from health services (33, 34), thus tactful conversations is of paramount importance given the significant psychological burden already existing in NAFLD (35).

## Limitations

The current study explores the prevalence, risk factors and longitudinal outcomes of poor weight perception in individuals with NAFLD. However, there are limitations to this study that should be acknowledged. Even though biopsy and imaging-based diagnosis of fatty liver is preferred in the setting of population studies, blood based non-invasive tests were employed for the diagnosis of hepatic steatosis in this study due to its practicality in data availability. Additionally, investigating physical activity levels in individuals through self-report questionnaires rather than monitor-based measures invites room for misclassification, recall and social desirability biases. Due to the retrospective nature of this study, liver-related events were not investigated due to the lack of available data in the present database used.

## Conclusions

The present analysis clearly demonstrates the positive association between poor weight perception and reduction in physical activity, which consequently leads to significantly greater risk of adverse events in individuals with NAFLD. For longitudinal outcomes to improve in individuals diagnosed with NAFLD, public health efforts must be directed to place greater emphasis on targeting awareness, knowledge, and attitudes with regards to weight perception, albeit in a tactful and respectful manner.

## Data availability statement

The original contributions presented in the study are included in the article/Supplementary material, further inquiries can be directed to the corresponding author/s.

## Author contributions

CN and MM: conceptualization and design. CF, CN, WL, YC, and KC: acquisition of data. CF, CN, WL, YC, KC, JX, DT, JQ, and CT: analysis and interpretation of data. CF, CN, WL, YC, KC, JX, DT, and ZH: writing—original draft. TK, SZ, BN, YD, NC, NS, MS, AS, MN, and MM: writing—review and editing. All authors approved the final version of the manuscript, including the authorship list and agreed to be accountable for all aspects of the work in ensuring that questions related to the accuracy or integrity of any part of the work are appropriately investigated and resolved.

## Conflict of interest

AS is President of Sanyal Biotechnology and has stock options in Genfit, Akarna, Tiziana, Indalo, Durect and Galmed. He has served as a consultant to Astra Zeneca, Nitto Denko, Enyo, Ardelyx, Conatus, Nimbus, Amarin, Salix, Tobira, Takeda, Jannsen, Gilead, Terns, Birdrock, Merck, Valeant, Boehringer-Ingelheim, Lilly, Hemoshear, Zafgen, Novartis, Novo Nordisk, Pfizer, Exhalenz, and Genfit. He has been an unpaid consultant to Intercept, Echosens, Immuron, Galectin, Fractyl, Syntlogic, Affimune, Chemomab, Zydus, Nordic Bioscience, Albireo, Prosciento, Surrozen, and Bristol Myers Squibb. His institution has received grant support from Gilead, Salix, Tobira, Bristol Myers, Shire, Intercept, Merck, Astra Zeneca, Malinckrodt, Cumberland, and Norvatis. He receives royalties from Elsevier and UptoDate. MN has been on the advisory board/consultant for 89BIO, Altimmune, Gilead, cohBar, Cytodyn, Intercept, Pfizer, Novo Nordisk, Blade, EchoSens, Fractyl, Madrgial, NorthSea, Prespecturm, Terns, Siemens and Roche diagnostic; MN has received research support from Allergan, BMS, Gilead, Galmed, Galectin, Genfit, Conatus, Enanta, Madrigal, Novartis, Pfizer, Shire, Viking, and Zydus. He is a shareholder or has stocks in Anaetos, Chrownwell, Ciema, Rivus Pharma, and Viking. The remaining authors declare that the research was conducted in the absence of any commercial or financial relationships that could be construed as a potential conflict of interest.

## Publisher's note

All claims expressed in this article are solely those of the authors and do not necessarily represent those of their affiliated organizations, or those of the publisher, the editors and the reviewers. Any product that may be evaluated in this article, or claim that may be made by its manufacturer, is not guaranteed or endorsed by the publisher.
